# Molecular Interplay Between PTEN, ARID1A, PD-L1, and MMR in Asian Ovarian Clear Cell Carcinoma: Implications for Immunotherapy Response and Patient Stratification

**DOI:** 10.3390/ijms26104915

**Published:** 2025-05-20

**Authors:** Chen-Hsuan Wu, Hao Lin, Yu-Che Ou, Hung-Chun Fu, Ming-Yu Yang, Chao-Cheng Huang

**Affiliations:** 1 Department of Obstetrics and Gynecology, Kaohsiung Chang Gung Memorial Hospital and Chang Gung University College of Medicine, Kaohsiung 833401, Taiwan; chenhsuan5@gmail.com (C.-H.W.); haolin423700@gmail.com (H.L.); ou4727@cgmh.org.tw (Y.-C.O.); allen133@cgmh.org.tw (H.-C.F.); 2 Graduate Institute of Clinical Medical Sciences, College of Medicine, Chang Gung University, Taoyuan 33302, Taiwan; yangmy@mail.cgu.edu.tw; 3 Department of Otolaryngology, Kaohsiung Chang Gung Memorial Hospital and Chang Gung University College of Medicine, Kaohsiung 833401, Taiwan; 4 Department of Anatomic Pathology, Kaohsiung Chang Gung Memorial Hospital and Chang Gung University College of Medicine, Kaohsiung 833401, Taiwan

**Keywords:** ovarian clear cell carcinoma, PD-L1, PTEN, ARID1A, mismatch repair

## Abstract

Ovarian clear cell carcinoma (OCCC) represents a distinct histological subtype with a high prevalence in Asian populations and poor chemotherapy response. This study investigated molecular interactions between phosphatase and tensin homolog (PTEN), AT-rich interactive domain 1A (ARID1A), programmed death-ligand 1 (PD-L1), and mismatch repair (MMR) proteins in Asian patients with OCCC. Immunohistochemical analysis was performed on tissue microarrays from 69 OCCC cases. The expression of PTEN, ARID1A, PD-L1, and four MMR proteins was evaluated alongside clinical data. A high prevalence of PTEN loss (78.3%) and ARID1A deficiency (48.8%), with PD-L1 expression in 26.1% and MMR deficiency in 10.1% of cases, was observed. All PD-L1-positive tumors demonstrated concurrent PTEN loss (*p* = 0.007). MMR deficiency was significantly associated with ARID1A loss (*p* = 0.049). PTEN loss correlated with worse progression-free survival (PFS) in early-stage disease (*p* = 0.039). PTEN and ARID1A alterations represent early pathogenic events in Asian OCCC, with PTEN loss significantly impacting PFS in early-stage disease. The correlation between PTEN loss and PD-L1 expression, alongside ARID1A-MMR deficiency association, provides insights into OCCC’s immunological landscape and therapeutic vulnerabilities.

## 1. Introduction

In Asia, ovarian clear cell carcinoma (OCCC) accounts for 20–25% of epithelial ovarian cancer (EOC), and unlike Western countries, where it comprises only 5–10% of cases, it represents the second or third most common histology of EOC [1]. Although OCCC usually presents at early stages with better survival compared to early-stage high-grade serous carcinoma, the disease typically shows a poor response to platinum-based treatment and other conventional chemotherapy regimens, with response rates below 30% in advanced or recurrent cases [2]. Recent studies have identified increased antioxidant capacity through NRF2 pathway activation as an important contributing factor to chemoresistance in ovarian cancer, highlighting the complex mechanisms underlying therapeutic resistance in this challenging malignancy [3].

Loss of phosphatase and tensin homolog (PTEN) expression related to genetic or epigenetic alterations is particularly common in OCCC, occurring in approximately 40–50% of cases, but its association with clinical characteristics and other immune-related biomarkers remains unclear [4]. PTEN loss has been linked to increased PI3K/AKT pathway activation, which may contribute to chemotherapy resistance and disease progression. Previous genome-wide sequencing studies have revealed that *PTEN* gene mutations frequently co-occur with *PIK3CA* gene mutations in OCCC, suggesting a synergistic effect in promoting oncogenesis through enhanced PI3K/AKT signaling [5].

AT-rich interactive-domain 1A (*ARID1A*) mutations, present in approximately 50–60% of OCCC cases, often result in loss of protein expression and dysregulation of chromatin remodeling [6]. The co-occurrence of ARID1A and PTEN alterations has been reported in about 30% of OCCC cases, potentially indicating a cooperative role in tumor development [7]. Mechanistic studies have demonstrated that ARID1A loss leads to impaired DNA damage repair and altered expression of genes involved in cell cycle regulation and apoptosis [8]. Recent studies have shown that immune checkpoint inhibitors may demonstrate efficacy in recurrent OCCC, with objective response rates of 15–20%, a response pattern not typically observed in other ovarian cancer subtypes [9]. Recent investigations have begun exploring the relationship between ARID1A expression and immunotherapy response, with preliminary data suggesting that ARID1A deficiency may enhance sensitivity to immune checkpoint blockade [10].

Given that mismatch repair (MMR) deficiency and programmed cell death-ligand 1 (PD-L1) expression serve as important biomarkers for immunotherapy response, with positive predictive values reported in multiple solid tumors, studies have shown that MMR deficiency occurs in approximately 2–3% of ovarian cancers and is associated with increased tumor mutational burden and enhanced response to immunotherapy [11]. Meanwhile, PD-L1 expression has been reported in 30–40% of OCCC cases and appears to correlate with tumor-infiltrating lymphocytes and improved survival outcomes [12].

Despite these individual findings, a significant knowledge gap exists in understanding the complex molecular interplay between these biomarkers, particularly in Asian OCCC populations. While previous studies have primarily focused on Western cohorts or examined these biomarkers in isolation, our study represents the first comprehensive analysis investigating the concurrent expression patterns and associations between PTEN, ARID1A, PD-L1, and MMR status specifically in an Asian OCCC cohort. Furthermore, whereas existing literature has largely explored these markers independently, our study uniquely examines their co-expression patterns and stage-specific relationships, providing novel insights into potential early pathogenic events and their prognostic significance.

Building on these findings, our study addresses critical gaps in OCCC research by (1) comprehensively profiling molecular biomarkers specifically in Asian patients with OCCC, in whom prevalence and patterns may differ significantly from those in Western populations; (2) systematically investigating the complex interrelationships among all four biomarkers (PTEN, ARID1A, PD-L1, and MMR) to understand their integrated role in OCCC biology; (3) analyzing their co-expression patterns across different disease stages to identify potential early pathogenic events and stage-specific molecular signatures; and (4) correlating key pathogenic markers (PTEN and ARID1A) with clinical outcomes to determine their prognostic value, while examining immune biomarkers (PD-L1 and MMR) for their potential therapeutic implications in immunotherapy selection. We aimed to provide a comprehensive analysis of these biomarkers in Asian patients with OCCC, exploring both their pathogenic significance and their utility in guiding personalized therapeutic strategies for this challenging malignancy.

## 2. Results

### 2.1. Clinicopathological Characteristics of Included Patients

**Figure 1:**
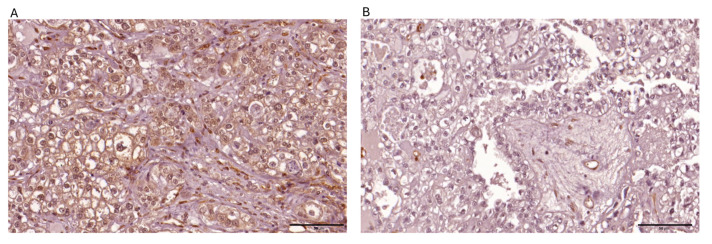
Representative images depicting immunohistochemical expression patterns of PTEN observed within ovarian clear cell carcinoma tissue specimens are presented: (**A**) strong cytoplasmic expression in >80% of tumor cells; (**B**) negative expression.

**Figure 2:**
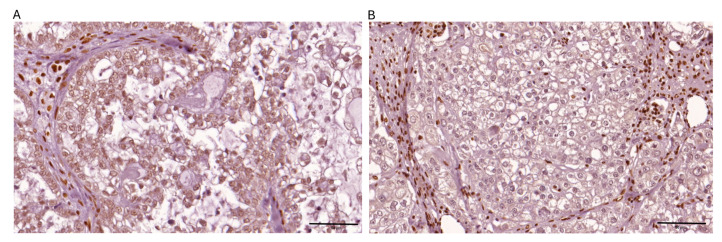
Representative images depicting immunohistochemical expression patterns of ARID1A observed within ovarian clear cell carcinoma tissue specimens are presented: (**A**) positive nuclear expression in >80% of tumor cells; (**B**) negative expression.

**Figure 3:**
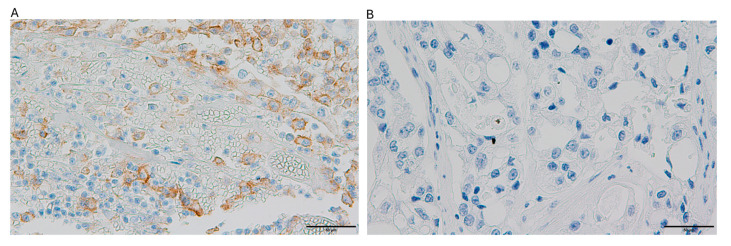
The images illustrate PD-L1 immunohistochemical staining patterns in ovarian clear cell carcinoma specimens: (**A**) tumor tissue demonstrating positive PD-L1 expression with a combined positive score (CPS) of 75%; (**B**) specimen exhibiting complete absence of PD-L1 expression.

**Figure 4:**
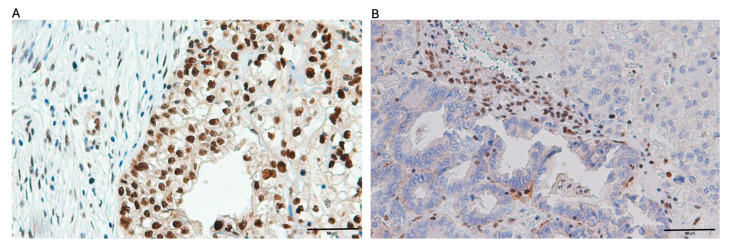
Illustrative microphotographs showing the immunohistochemical staining patterns of mismatch repair (MMR) proteins in ovarian clear cell carcinoma tissue samples: (**A**) specimen displaying positive MLH1 protein expression; (**B**) tissue section demonstrating absence of MSH2 protein expression.

Among the 69 ovarian clear cell carcinoma cases analyzed, PTEN expression was negative in 78.3% (54/69) of cases, while ARID1A loss was observed in 48.8% (39/69) of cases. PD-L1 expression was positive in 26.1% (18/69) of cases. dMMR was observed in 10.1% (*n* = 7) of cases, while 89.9% (*n* = 62) maintained pMMR function. PD-L1 expression was positive in 26.1% (*n* = 18) of tumors and negative in 73.9% (*n* = 51) (Table 1). The representative images depicting immunohistochemical expression patterns of the four targeted biomarkers are presented in Figure 1, Figure 2, Figure 3 and Figure 4.

### 2.2. Expression Patterns According to Disease Stage and Molecular Interrelationships Between Biomarkers

We evaluated MMR status and PD-L1 expressions alongside other clinicopathological characteristics. The mean age was comparable across both MMR and PD-L1 subgroups (approximately 50 years). Most patients presented with early-stage disease (FIGO stages I/II). Notably, there was a significant association between ARID1A expression and MMR status, with ARID1A loss being significantly more frequent in dMMR tumors (85.7% vs. 43.5% in pMMR tumors, *p* = 0.049). Strikingly, all dMMR tumors (100%) showed PTEN loss, compared to 75.8% of pMMR tumors, although this difference did not reach statistical significance (*p* = 0.333). A striking finding in our study was that all PD-L1-positive tumors (100%) demonstrated PTEN loss, which was significantly higher than in PD-L1-negative tumors (70.6%, *p* = 0.007). There was no significant association between PD-L1 expression and MMR status (*p* = 0.667), nor was there any significant correlation between these molecular features and platinum sensitivity or FIGO stage (Table 2).

### 2.3. Co-Expression Analysis of ARID1A and PTEN According to Disease Stage

The concurrent expression patterns of ARID1A and PTEN were analyzed, revealing four distinct groups: ARID1A+/PTEN+ (14.5%), ARID1A+/PTEN- (37.7%), ARID1A-/PTEN+ (7.2%), and ARID1A-/PTEN- (40.6%). In early-stage disease, the distribution shifted notably, with ARID1A-/PTEN- being the predominant pattern (45.4%), followed by ARID1A+/PTEN- (36.4%), ARID1A-/PTEN+ (11.3%), and ARID1A+/PTEN+ (6.8%). In advanced-stage disease, a different pattern emerged, with ARID1A+/PTEN- being most common (41.7%), followed by ARID1A-/PTEN- (33.3%) and ARID1A+/PTEN+ (25%), while no cases showed the ARID1A-/PTEN+ pattern (*p* = 0.066) (Table 3). These findings demonstrate the heterogeneous molecular profile of ovarian clear cell carcinoma in our patient population, with a predominance of early-stage disease and a high frequency of PTEN loss and ARID1A alteration.

### 2.4. Treatment Response and Survival Analysis

**Figure 5:**
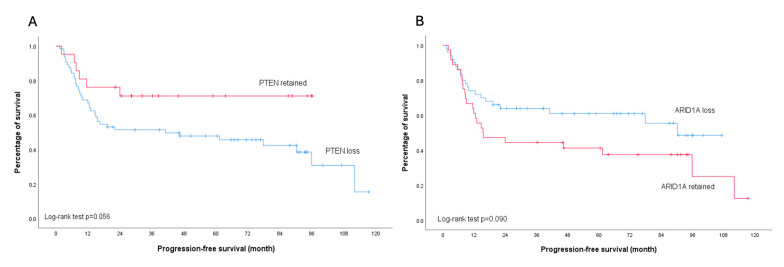
Kaplan–Meier analysis illustrates progression-free survival outcomes across all stages of ovarian clear cell carcinoma. (**A**) Stratified survival curves comparing patients with PTEN protein retention (red line) versus PTEN loss (blue line); log-rank test *p* = 0.056. (**B**) Stratified survival curves comparing patients with ARID1A protein retention (red line) versus ARID1A loss (blue line); log-rank test *p* = 0.090.

**Figure 6:**
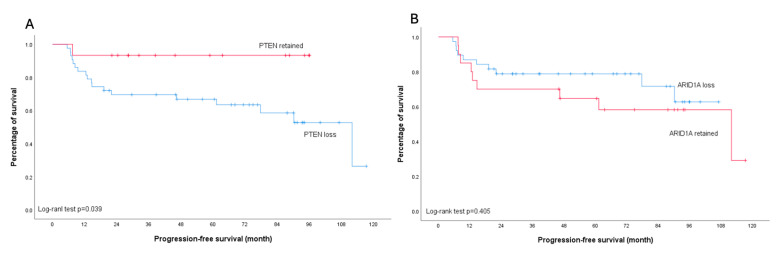
Kaplan–Meier curves illustrating progression-free survival in early-stage ovarian clear cell carcinoma patients. (**A**) Stratification by PTEN status: retained (red line) versus loss (blue line); log-rank test *p* = 0.039. (**B**) Stratification by ARID1A status: retained (red line) versus loss (blue line); log-rank test *p* = 0.405.

None of the analyzed molecular markers showed a significant association with platinum sensitivity (all *p* > 0.05) (Table 2). Regarding survival outcomes, PTEN loss was associated with worse PFS, particularly in early-stage disease (*p* = 0.039). This trend was also observed in the overall cohort, though with borderline significance (*p* = 0.056). In contrast, patients with ARID1A loss demonstrated a trend toward improved PFS compared to those with retained ARID1A expression across all stages, although this did not reach statistical significance (*p* = 0.090). Importantly, subgroup analysis of early-stage disease revealed no difference in PFS between patients with ARID1A loss and those with retained ARID1A expression (*p* = 0.405) (Figure 5 and Figure 6).

## 3. Discussion

This study demonstrated the high prevalence of the alteration of PTEN and ARID1A expression as well as the low percentage of deficient MMR expression in OCCC. Notably, 26.1% of all OCCC cases showed positive PD-L1 expression, and all these PD-L1-positive cases demonstrated concurrent PTEN loss. Additionally, we identified a significant association between MMR deficiency and ARID1A loss, with 85.7% of ARID1A-deficient cases showing MMR loss compared to only 14.3% in ARID1A-preserved cases. Most importantly, PTEN loss correlated significantly with PFS, particularly in early-stage disease. These findings reveal distinct molecular patterns that warrant further investigation, particularly in the context of Asian OCCC populations.

### 3.1. Distinct Molecular Characteristics of ARID1A and PTEN Expression Patterns in Asian OCCC Cohort: Early Alterations and Stage-Specific Dynamics

OCCC demonstrates distinct geographical distribution patterns, representing 5% to 13% of epithelial ovarian cancer in Western populations and accounting for up to 20–25% in East Asian countries [13]. Our finding of a high PTEN loss rate (78.3%) in this Asian cohort substantially exceeds the previously reported 40–60% in Western populations [14]. This remarkable difference aligns with recent studies highlighting population-specific molecular profiles in OCCC [15]. Particularly noteworthy in our cohort is the co-occurrence pattern of PTEN and ARID1A loss, with 46.5% of early-stage cases showing concurrent loss, while only 7.0% maintained expression of both markers. These findings further corroborate the theory proposed in previous studies [16] and indicate that these molecular alterations represent fundamental early events in the pathogenesis of OCCC [17,18].

Despite not reaching statistical significance (*p* = 0.066), possibly due to limited advanced-stage cases (*n* = 24), we observed intriguing patterns in ARID1A/PTEN expression across disease stages. The unexpectedly higher proportion of A+/P+ cases in advanced versus early stage (25.0% vs. 6.8%) suggests complex underlying mechanisms. This molecular complexity in advanced OCCC has been well-documented by Bolton, K.L. et al., who identified distinct molecular subgroups with different therapeutic vulnerabilities [19]. These findings reflect tumor heterogeneity, with advanced tumors maintaining protein expression while harboring alternative pathway alterations. This observation aligns with Chao, A. et al., who demonstrated that advanced OCCCs often develop resistance mechanisms through the activation of alternative pathways, potentially indicating dynamic protein expression changes through disease progression [20]. These findings challenge traditional views of tumor suppressor roles and suggest possible the involvement of tumor microenvironment interactions or post-translational modifications in OCCC progression. Furthermore, Wijaya, S.T. et al. reported improved response rates in patients with advanced OCCC treated with combination targeted therapies based on comprehensive molecular profiling, supporting the need for larger cohort studies to validate these stage-specific molecular patterns and their therapeutic implications [21].

### 3.2. PTEN Loss as an Early Prognostic Indicator in OCCC: Implications for Stage-Specific Therapeutic Strategies

In our cohort, PTEN loss demonstrated a significant correlation with PFS, particularly in early-stage OCCC. This finding aligns with previous studies suggesting PTEN’s crucial role in tumor progression through the PI3K/AKT/mTOR pathway [4,22]. Previous molecular studies have revealed that PTEN loss leads to constitutive activation of the PI3K/AKT pathway, resulting in enhanced cell survival and proliferation [23]. Additionally, demonstrated that PTEN-deficient OCCC cells had been demonstrated to exhibit increased resistance to platinum-based chemotherapy, potentially explaining the poor treatment outcomes observed in these patients [24]. Moreover, despite both ARID1A and PTEN being involved in OCCC pathogenesis, they exhibit distinct prognostic value, particularly in early-stage disease, in which PTEN may be a more relevant prognostic indicator, while ARID1A expression status has minimal impact on survival outcomes. The particularly strong correlation in early-stage disease suggests that PTEN loss might be an early event in OCCC progression. This hypothesis is supported by previous work reporting PTEN alterations in endometriosis-associated OCCC precursor lesions [25]. Furthermore, integrated genomic analyses also revealed that PTEN loss often precedes other molecular alterations in OCCC development, highlighting its potential value as an early prognostic marker [20]. These stage-specific patterns of PTEN loss and their differential impact on survival suggest the need for distinct therapeutic approaches in early versus advanced OCCC. Beyond individual marker analysis, understanding the complex interactions between these molecular alterations is crucial for developing effective therapeutic strategies. The unexpected associations between different molecular markers in our study suggest intricate pathway crosstalk that may have significant implications for treatment approaches.

### 3.3. MMR Deficiency and PI3K–AKT–mTOR Pathway: Molecular Interplay in Immune Evasion

In our cohort, all tumors with dMMR (100%) exhibited concurrent PTEN protein loss, compared to 75.8% of PTEN loss in pMMR tumors. Although this association did not reach statistical significance, likely due to the limited number of dMMR cases, the consistent co-occurrence of dMMR and PTEN loss warrants further attention. This finding aligns with recent research demonstrating potential molecular crosstalk between the PI3K–AKT–mTOR pathway and mismatch repair proteins. Wang et al. found that in dMMR gastric adenocarcinoma, a higher mutation burden in the PI3K–AKT–mTOR pathway correlated with reduced immune cell infiltration and inferior response to immune checkpoint inhibitors [26]. This suggests that alterations in the PI3K–AKT–mTOR pathway might be one of the mechanisms underlying immune evasion and primary resistance to immunotherapy in dMMR tumors.

### 3.4. ARID1A-MMR Relationship: Implications for DNA Repair and Immunotherapy Response

Emerging research has uncovered critical information about OCCC’s immunological characteristics and survival outcomes, paving the way for immune-based therapeutic strategies while revealing distinct immune patterns that affect patient prognosis [27]. Our analysis revealed a significant association between ARID1A loss and MMR deficiency, with 18.6% of ARID1A-loss tumors exhibiting MMR loss, compared to only 2.8% in ARID1A-preserved tumors. This finding aligns with previous studies suggesting that ARID1A, a component of the SWI/SNF chromatin remodeling complex, plays a crucial role in DNA damage response and repair mechanisms [28]. While this relationship has been underexplored in OCCC, mechanistic studies in other cancers have demonstrated that ARID1A deficiency impairs MMR protein expression through disruption of MLH1 transcription and compromises DNA mismatch repair through SWI/SNF complex-mediated chromatin remodeling [29,30,31,32]. These findings suggest a conserved mechanistic link between ARID1A loss and MMR deficiency that may have therapeutic implications, as both ARID1A-deficient tumors and MMR-deficient tumors have shown sensitivity to immunotherapy approaches. Although our cohort showed no significant difference in PD-L1 expression between ARID1A-loss and ARID1A-preserved tumors, the MMR status correlation warrants further investigation into immunotherapeutic approaches for ARID1A-deficient cancers.

### 3.5. PTEN-PD-L1 Axis: Targeting Immune Checkpoint Pathways in OCCC

A striking finding in our study was the perfect correlation between PTEN loss and PD-L1 expression, with all PD-L1-positive cases showing PTEN loss. This clinical observation validates recent preclinical studies demonstrating that PTEN loss directly upregulates PD-L1 expression through PI3K/AKT/mTOR pathway activation and specific transcriptional regulators [33,34]. In addition, the influence of PTEN deficiency and PI3K activation on T cell-driven antitumor immunity was examined using a preclinical melanoma model [35], and recent preclinical studies have also shown synergistic effects when combining PI3K inhibitors with immune checkpoint blockade in PTEN-deficient models [36]. More and more studies have reported changes in *PTEN* and the interplay with other genes studied in mouse models of prostate cancer has been shown to collectively shape the immune cell content and expression of immunosuppressive markers in the tumor microenvironment [37,38], providing a strong rationale for investigating similar strategies in patients with OCCC with this molecular profile.

Collectively, these molecular interactions highlight complex interconnections between chromatin remodeling, DNA repair, and immune regulation pathways in OCCC. While platinum sensitivity did not significantly differ between PD-L1-positive and PD-L1-negative tumors, the complete concordance between PD-L1 positivity and PTEN alteration suggests that PTEN status could serve as a surrogate biomarker for potential responsiveness to PD-1/PD-L1 inhibitors. PTEN, a critical negative regulator of the PI3K–AKT–mTOR pathway, appears to be a central player in immune evasion through its direct impact on PD-L1 expression. These findings further highlight the importance of assessing both PTEN and PD-L1 status when considering immunotherapeutic strategies in OCCC, as dual targeting of the PI3K/AKT pathway and immune checkpoints may provide synergistic effects in tumors with these molecular features. Larger studies are needed to validate these observations and determine whether these molecular signatures define distinct OCCC subtypes with unique therapeutic vulnerabilities that could optimize treatment selection and improve outcomes in this challenging malignancy.

### 3.6. Limitations

Despite the significant findings, several limitations of this study warrant consideration. First, the retrospective nature and relatively modest sample size (*n* = 69) may limit the statistical power, particularly for subgroup analyses of advanced-stage disease. Second, our reliance on immunohistochemistry for protein expression assessment, while clinically applicable, does not capture all genetic or epigenetic alterations that might affect these pathways. Specifically, post-translational modifications and functional pathway activation cannot be fully evaluated through IHC analysis alone. Third, our tissue microarray approach, although including three cores per tumor, may not fully account for intratumoral heterogeneity known to exist in OCCC. Further studies with larger cohorts and comprehensive molecular profiling are needed to confirm these associations and their therapeutic implications.

## 4. Materials and Methods

This study was approved by the Institutional Review Board of Chang Gung Memorial Hospital (CGMH), Taiwan, and patient informed consent was waived (number 102-1076B, 105-0992C). We confirm that all experiments were performed in accordance with relevant guidelines and regulations.

### 4.1. Patients and Settings

Cases and their pathology data were retrospectively collected, including all stages of OCCC diagnosed at Kaohsiung Chang Memorial Hospital between 2008 and 2013. Diagnosis was based on the conventional histomorphological examination and ancillary immunohistochemical (IHC) study, if necessary, and all cases were re-reviewed by 2 surgical pathologists to confirm the diagnosis. Tumor staging was performed according to the 2014 International Federation of Gynecology and Obstetrics (FIGO) classification. All patients were primarily treated with staging surgery or debulking surgery followed by at least 3 cycles of adjuvant platinum-based combination chemotherapy (paclitaxel 175 mg/m^2^ and carboplatin area under the curve [AUC] 5 or CDDP 75 mg/m^2^ and cyclophosphamide 750 mg/m^2^) according to the disease stage.

### 4.2. Assembling of Tissue Array

The paraffin tissue blocks were assembled into tissue arrays using an automated tissue array, TMA Grand Master (3DHISTECH Ltd., Budapest, Hungary). Briefly, the selected tumor areas were marked on the hematoxylin and eosin-stained slides to match the corresponding areas on the paraffin tissue blocks. Considering the tissue heterogeneity, three tissue cores for each tumor were obtained using a tissue cylinder of 1.5 mm in diameter to assemble the tissue array blocks. In addition, leiomyoma tissue was included in each tissue array block for orientation and as external controls.

### 4.3. Immunohistochemistry Study of Targeted Biomarkers

IHC analysis was performed on 3 μm sections sliced from the tissue array blocks. Following deparaffinization and rehydration of the tissue’s sections, antigen retrieval was performed at 100 °C for 15 min with 1X citrate buffer, pH 6.0 (Thermo Fisher Scientific, Waltham, MA, USA; cat. no. AP-9003-500). Endogenous peroxidase was blocked with 3% H_2_O_2_ for 15 min. All patients included had IHC testing for ARID1A, PTEN, and MMR, and PD-L1 expression with MMR was evaluated through the assessment of 4 proteins: MSH2, MSH6, MLH1, and PMS2. Primary antibodies against ARID1A, PTEN, four MMR proteins (MLH1, MSH2, MSH6, and PMS2), and PD-L1 were applied, as detailed in Table 4. All primary antibodies were qualified by the Nordic IHC Quality Control (NordiQC, Aalborg, Denmark) scientific organization, except for MSH6 antibody (Genemed, South San Francisco, CA, USA; clone 024). Primary antibody detection was carried out using a polymer system (Thermo Fisher Scientific, Waltham, MA, USA; cat. no. TL-125-QHL). Staining development was achieved by incubation with DAB and DAB Enhancer (Thermo Fisher Scientific, Waltham, MA, USA; cat. no. TA-125-QHDX).

### 4.4. Definition for ARID1A and PTEN Expressions

Two pathologists independently examined all the sections of the tumor tissue array. Immunostainings of ARID1A protein expression and PTEN protein in tumor cells were scored following a semiquantitative method. The positive immunostaining was shown in the cytoplasm for PTEN and in the nucleus for ARID1A-encoded protein, BAF250A. The staining intensity in tumor cells was compared to that detected in the adjacent stromal cells, which were used as positive controls. The intensity of BAF250A and PTEN immunostainings was scored as 0 (no staining), 1 (weaker staining than that of stromal cells), and 2 (equal to that of stromal cells). Only a score of 2 for intensity was defined as positive. The percentage of positive tumor cells was further scored as 0 (0%), 1+ (≤10%), 2+ (11–50%), 3+ (51–80%), and 4+ (>80%) using the scoring system proposed by Samartzis et al. [39]. Tumor tissue was classified as having downregulation or loss of ARID1A or PTEN expression when either the percentage of positive tumor cells was scored as 3+ or below (corresponding to ≤80% positive tumor cells) or when the staining intensity was scored as 0 or 1.

### 4.5. Definition of Positive PD-L1 and Deficient Expression of MMR Proteins

Internal positive controls consisted of non-cancerous ovarian and stroma cells. With appropriate internal positive controls, nuclear staining in tumor cells was considered to be a normal expression (proficiency) of MMR proteins, while a total absence of nuclear staining was interpreted as a loss of expression (deficiency). Tumor MMR status was assessed and determined by the IHC expression of four MMR proteins (MLH1, MSH2, MSH6, and PMS2); the definition of deficient MMR (dMMR) was the loss of at least one of these four proteins, while proficient MMR (pMMR) was defined as the intact expression of all four MMR proteins. An anti-PD-L1 22C3 IHC assay was used to evaluate the tumor PD-L1 expression. For PD-L1 expression evaluation, we utilized the combined positive score (CPS) method. The CPS was calculated as the number of PD-L1 staining cells (including tumor cells, lymphocytes, and macrophages) divided by the total number of viable tumor cells, multiplied by 100. A CPS of ≥1 was defined as PD-L1-positive. All slides were independently assessed by two pathologists blinded to clinical outcomes, with discrepancies resolved by consensus review.

### 4.6. Statistical Analysis

Progression-free survival (PFS) was calculated from the date of initial diagnosis to the date of first disease progression or death from any cause. Comparisons between categorical variables were conducted using chi-square tests or Fisher’s exact tests when appropriate. We estimated survival probabilities using the Kaplan–Meier method and evaluated differences between groups with log-rank tests. All statistical analyses were performed using IBM SPSS Statistics software version 26 (IBM Corporation, Armonk, NY, USA), with *p* < 0.05 considered statistically significant.

## 5. Conclusions

Ovarian clear cell carcinoma presents unique challenges in Asian populations, with distinct molecular features that demand tailored therapeutic approaches. This study addresses the critical need for population-specific biomarker profiling by demonstrating that Asian OCCC exhibits significantly higher PTEN loss rates (78.3%) than previously reported in Western cohorts. Our comprehensive analysis of four key molecular markers provides a framework for personalized treatment strategies in this challenging malignancy.

The integration of PTEN, ARID1A, MMR, and PD-L1 profiling enables a more nuanced approach to patient management. Early-stage patients with PTEN loss face worse progression-free survival and may benefit from intensified surveillance and aggressive adjuvant therapy. The perfect concordance between PTEN loss and PD-L1 expression identifies a specific patient subset ideally suited for combination PI3K inhibitor and immunotherapy approaches. Meanwhile, the ARID1A-MMR relationship reveals opportunities for chromatin remodeling-targeted treatments in immunotherapy candidates.

Moving forward, the clinical implementation of this molecular framework requires prospective validation through dedicated Asian OCCC cohort studies and biomarker-stratified clinical trials. Healthcare systems should prioritize the development of companion diagnostics that simultaneously assess these four biomarkers to streamline patient stratification. By embracing this molecularly informed paradigm, we can transform the treatment landscape for Asian OCCC patients, shifting from one-size-fits-all chemotherapy toward precision oncology approaches that address the unique biological characteristics of this disease subtype.

## Figures and Tables

**Table 1:** Clinicopathological characteristics of all patients (*n* = 69).

Characteristic	Value
Age, mean (SD)	50.6 (9.2)
Follow-up, months, median (range)	50 (5–132)
FIGO stage, n (%)	
I	36 (52.2)
II	8 (11.6)
III	18 (26.1)
IV	6 (8.7)
Unknown	1 (1.4)
Concurrent endometriosis	
Yes	15 (21.7)
No	54 (78.3)
ARID1A expression, n (%)	
Intact	41 (51.2)
Altered	39 (48.8)
PTEN expression, n (%)	
Strong	15 (21.7)
Weak or loss	54 (78.3)
MMR status, n (%)	
Proficient	62 (89.9)
Deficient	7 (10.1)
PD-L1 expression, n (%)	
Positive	18 (26.1.)
Negative	51 (73.9)

Note: FIGO: International Federation of Gynecology and Obstetrics; MMR: mismatch repair.

**Table 2:** Demographic and molecular characteristics of patients with ovarian CCC by MMR status and PD-L1 expression.

Total Number *n* = 69, 100%	pMMR *n* = 62, 89.9%	dMMR- *n* = 7, 10.1%	*p*-Value	PD-L1 (+) *n* = 18, 26.1%	PD-L1 (-) *n* = 51, 73.9%	*p*-Value
Variables	*n* (%)			*n* (%)		
Age Mean (SD)	50.6 (8.89)	51.0 (12.3)	0.903	50.2 (7.67)	50.8 (9.73)	0.835
FIGO Stage * I/II III/IV	38 (63.3) 22 (36.7)	5 (71.4) 2 (28.6)	1.000	11(61.1) 7 (38.9)	32 (65.3) 17 (34.7)	0.751
Platinum sensitivity ** Sensitive Resistant	42 (75.0) 14 (25.0)	4 (66.7) 2 (33.3)	0.643	14 (77.8) 4 (22.2)	32 (72.7) 12 (27.3)	0.760
ARID1A expression Retained Loss	35 (56.5) 27 (43.5)	1 (14.3) 6 (85.7)	**0.049**	8 (44.4) 10(55.6)	28 (54.9) 23 (45.1)	0.445
PTEN expression Retained Loss	15 (24.2) 47 (75.8)	0 (0) 7 (100)	0.333	0 (0) 18 (100)	15 (29.4) 36 (70.6)	**0.007**
PD-L1 expression Positive Negative	17 (27.4) 45 (72.6)	1 (14.3) 6 (85.7)	0.667	NA	NA	NA
MMR status Proficient Deficient	NA	NA	NA	17 (94.4) 1 (5.6)	45 (88.2) 6 (11.8)	0.667

Note: PD-L1 (+): positive PD-L1 expression if CPS ≥ 1; PD-L1 (-): negative PD-L1 expression if CPS < 1; SD: standard deviation; *: excluded 1 case with unknown stage; **: comprised 62 patients with complete data available for drug sensitivity analysis; FIGO: International Federation of Gynecology and Obstetrics; pMMR: proficient mismatch repair; dMMR: deficient mismatch repair.

**Table 3:** Distribution of ARID1A and PTEN expression patterns in patients with early- and advanced-stage ovarian CCC.

Stage	A+/P+	A+/P-	A-/P+	A-/P-	*p*-Value
Early (*n* = 44)	3 (6.8)	16 (36.4)	5 (11.4)	20 (45.4)	1.000
Advanced (*n* = 24)	6 (25.0)	10 (41.7)	0 (0.0)	8 (33.3)	0.066
Total * (*n* = 69)	10 (14.5)	26 (37.7)	5 (7.2)	28 (40.36)	0.204

Note: Data are presented as *n* (%). A+: ARID1A expression retained; A-: ARID1A expression lost; P+: PTEN expression retained; P-: PTEN expression lost; * included 1 case with unknown stage.

**Table 4:** Primary antibodies used in immunohistochemical Analysis.

Antibody	Clone	Dilution	Manufacturer
ARID1A	-	1:2000	Sigma (HPA005456)
PTEN	-	1:200	Cell Signaling (#9559)
MLH1	GM011	1:50	Genemed
MSH2	G219-1129	1:100	ZETA
MSH6	GM024	1:100	Genemed
PMS2	A16-4	1:100	BD Biosciences
PD-L1	22C3	1:50	Agilent (GE00621-2)

Note: Manufacturer locations: Sigma (St. Louis, MO, USA); Cell Signaling (Danvers, MA, USA); Genemed (South San Francisco, CA, USA); ZETA Corporation (Sierra Madre, CA, USA); BD Biosciences (San Jose, CA, USA); Agilent Technologies (Santa Clara, CA, USA).

## Data Availability

All data from this study are available upon reasonable request from the corresponding author.

## Abbreviations

The following abbreviations are used in this manuscript:

OCCC	Ovarian clear cell carcinoma
PTEN	Phosphatase and tensin homolog
ARID1A	AT-rich interactive domain 1A
PD-L1	Programmed cell death-ligand 1
MMR	Mismatch repair
IHC	Immunohistochemistry
dMMR	Deficient mismatch repair
pMMR	Proficient mismatch repair
CPS	Combined positive score
PFS	Progression-free survival
